# Where has all the influenza gone? The impact of COVID-19 on the circulation of influenza and other respiratory viruses, Australia, March to September 2020

**DOI:** 10.2807/1560-7917.ES.2020.25.47.2001847

**Published:** 2020-11-26

**Authors:** Sheena G Sullivan, Sandra Carlson, Allen C Cheng, Monique BN Chilver, Dominic E Dwyer, Melissa Irwin, Jen Kok, Kristine Macartney, Jennifer MacLachlan, Cara Minney-Smith, David Smith, Nigel Stocks, Janette Taylor, Ian G Barr

**Affiliations:** 1 WHO Collaborating Centre for Reference and Research on Influenza, Royal Melbourne Hospital, and Doherty Department, University of Melbourne, at the Peter Doherty Institute for Infection and Immunity, Melbourne, Australia; 2 FluTracking, Hunter New England Population Health, Newcastle, Australia; 3 School of Public Health and Preventive Medicine, Monash University, Melbourne, Australia; 4 Department of Infectious Diseases, Alfred Health, and Central Clinical School, Monash University, Melbourne, Australia; 5 Discipline of General Practice, University of Adelaide, Adelaide, Australia; 6 Centre for Infectious Diseases and Microbiology Laboratory Services, New South Wales Health Pathology - Institute of Clinical Pathology and Medical Research, Westmead Hospital, Westmead, Australia; 7 Rapid Surveillance, Centre for Epidemiology and Evidence, NSW Ministry of Health, Sydney, Australia; 8 National Centre for Immunisation Research and Surveillance and The Children’s Hospital Westmead, Sydney, Australia; 9 Faculty of Medicine and Health, The University of Sydney, Sydney, Australia; 10 WHO Collaborating Centre for Viral Hepatitis, Royal Melbourne Hospital at the Peter Doherty Institute for Infection and Immunity, Melbourne, Australia; 11 PathWest Laboratory Medicine WA, Nedlands, Australia; 12 Faculty of Health and Medical Sciences, University of Western Australia, Nedlands, Australia; 13 WHO Collaborating Centre for Reference and Research on Influenza, Royal Melbourne Hospital, and Department of Microbiology and Immunology, University of Melbourne, at the Peter Doherty Institute for Infection and Immunity, Melbourne, Australia

**Keywords:** influenza, respiratory syncytial virus, rhinoviruses, travel restrictions, non-pharmaceutical interventions

## Abstract

The coronavirus disease pandemic was declared in March 2020, as the southern hemisphere’s winter approached. Australia expected co-circulation of severe acute respiratory syndrome coronavirus 2, influenza and other seasonal respiratory viruses. However, influenza notifications were 7,029 (March–September) compared with an average 149,832 for the same period in 2015–2109, despite substantial testing. Restrictions on movement within and into Australia may have temporarily eliminated influenza. Other respiratory pathogens also showed remarkably changed activity in 2020.

The World Health Organization (WHO) declared a coronavirus disease (COVID-19) pandemic on 11 March 2020, as southern hemisphere countries prepared for their usual winter respiratory pathogen epidemics. The announcement prompted concern that severe acute respiratory syndrome coronavirus 2 (SARS-CoV-2), influenza and other respiratory viruses might co-circulate, straining and possibly overwhelming healthcare systems. In Australia, these fears were not realised and—in contrast to expectations—influenza activity was at an all-time low during the southern hemisphere’s 2020 winter. Here, we describe the decreased activity of influenza and other respiratory pathogens in Australia and the measures that have likely contributed to their decline.

## Pandemic mitigation measures and influenza and COVID-19 notifications

**Figure 1:**
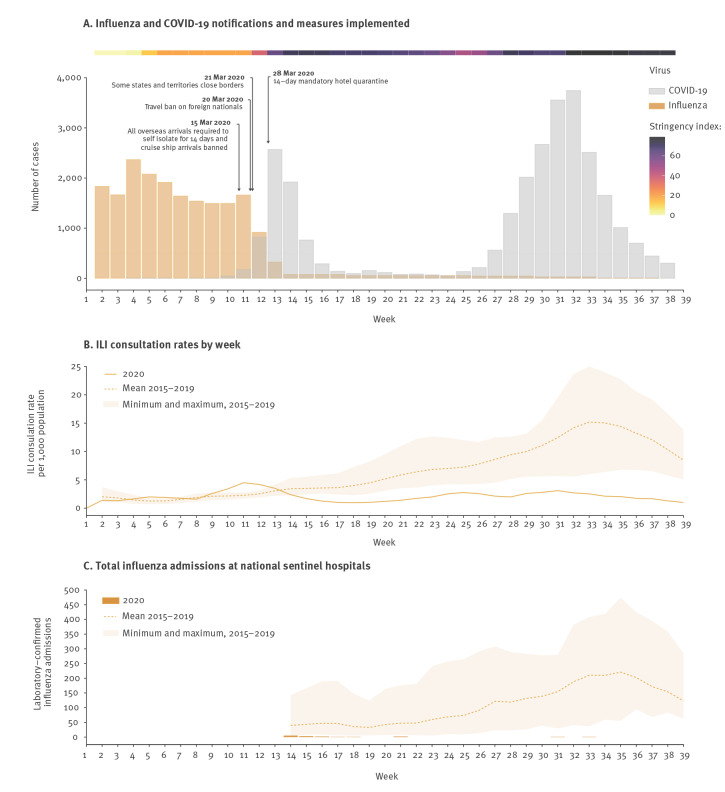
Surveillance data on (A) influenza and COVID-19 notifications and measures implemented, (B) ILI consultation rates by week and (C) total influenza admissions per week at national sentinel hospitals, Australia, as at 30 September 2020

COVID-19: coronavirus disease; ILI: influenza-like illness.

Key government measures to restrict movement and mixing are displayed in Figure 1A, with severity indicated using the Oxford Stringency Index [1]. Like many other countries, COVID-19 mitigation measures implemented in Australia included working from home, limits on types of indoor and outdoor social gatherings (such as meals, organised sport and religious services) and numbers of attendees, visitor restrictions in hospitals and residential long-term care facilities, increased use of hand sanitisers and other hygiene measures, and physical distancing [2]. The duration and intensity of these measures varied nationwide, with Victoria the only jurisdiction to require persons (aged >12 years) to wear face masks in public and to carry out extended school closures. Domestic inter-jurisdictional borders were closed by five of eight states and territories in late March. National borders were incrementally closed to foreign nationals with all returned travellers required to self-isolate for 14 days in hotel quarantine from 28 March [2].

Panel A: Influenza notifications are based on monthly notifications with weekly variation inferred from weekly counts for New South Wales. The Oxford Stringency Index indicates the severity of government restrictions [1]. Source: National Notifiable Diseases Surveillance System and www.covid19data.com.au.

Panel B: 3-week moving average. Source: Australian Sentinel Practices Research Network.

Panel C: Virological testing only detected one influenza virus since April. Source: Influenza Complications Alert Network.

Influenza is notifiable in Australia and notifications averaged around 2,000 cases per week from January to March 2020, with a precipitous drop between mid-March (week 11) and mid-April (week 14), when travel and work restrictions were introduced (Figure 1A). Concurrently, COVID-19 notifications began to increase, peaking in April 2020 at levels comparable to the inter-seasonal influenza activity seen from January to February. Physical, but not travel, restrictions were somewhat relaxed from May to June for most of the country (Figure 1A [2];). A second COVID-19 epidemic was experienced in the state of Victoria only, in July 2020, leading to the re-implementation of strict control measures and accounting for the vast majority of cases notified (94%) from July to September. However, the subsequent relaxation of restrictions across most of the country has not been accompanied by increases in influenza notifications during the normal influenza season (approximately weeks 18–44).

## Sentinel surveillance

In Australia, influenza-like illness (ILI) surveillance in primary care is conducted year-round to capture seasonal and inter-seasonal influenza patterns, as well as activity in the tropical northern region and infections in individuals following international travel [3]. ILI surveillance usually shows clear seasonality, with consultation rates highest in July and August (Figure 1B). However, following considerable influenza activity in 2019 [4], ILI consultation rates were well below average in 2020. ILI sentinel surveillance detected just one influenza and three respiratory syncytial virus (RSV) cases among 587 swabs tested from April to September.

National sentinel hospitalisation data (20 sites, FluCAN-PAEDS network [5]) reflects a similar steep decline in influenza-associated hospitalisations (Figure 1C). There were also far fewer influenza-attributable deaths reported nationally (36 deaths from January to September 2020, compared with 812 deaths for the same period in 2019 [6]) and, since April, deaths attributed to respiratory causes have been below historical averages [7].

Consistency in primary care, hospitalisation and mortality data all suggest that these declines cannot be entirely attributed to changes in healthcare-seeking behaviour.

## Virological surveillance

**Figure 2:**
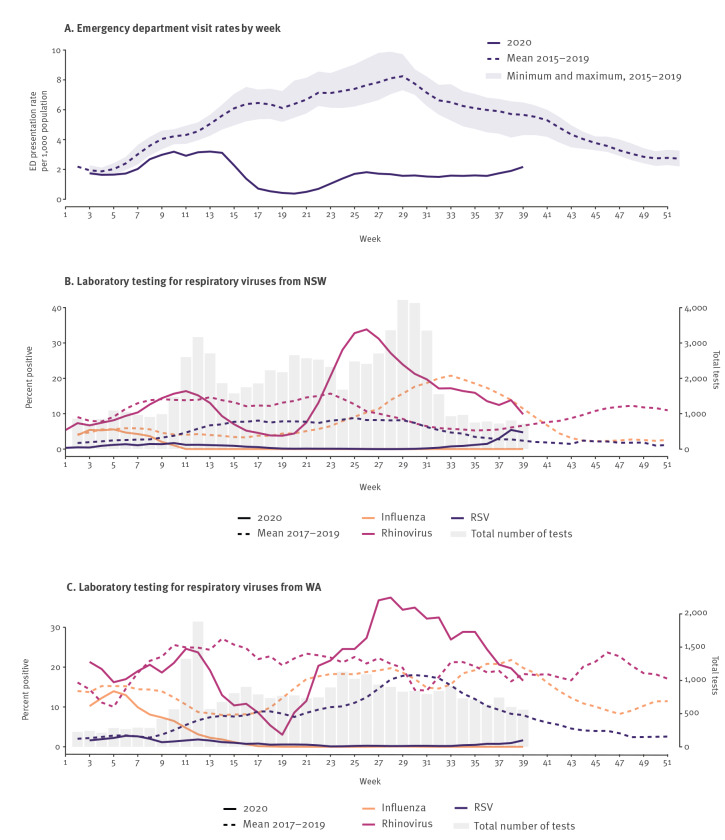
Selected surveillance data on (A) emergency department visit rates by week and weekly laboratory testing for respiratory viruses from (B) NSW and (C) WA, Australia, January–September 2020

ED: emergency department; NSW: New South Wales; RSV: respiratory syncytial virus; WA: Western Australia.

Real-time RT-PCR testing capacity in Australia considerably expanded in 2020 with the introduction of, and substantial demand for, SARS-CoV-2 testing. Initially, COVID-19 disrupted testing for non–SARS-CoV-2 respiratory viruses at some laboratories. However, even after public and private laboratories increased testing capacity for other respiratory viruses (by up to a three-fold increase in some jurisdictions (data not shown)), influenza virus detections remained exceedingly low [6]. Data from New South Wales (NSW) and Western Australia (WA) showed major reductions in the weekly percentages of tests positive for either influenza viruses or RSV (Figure 2B-C). Bronchiolitis surveillance from NSW emergency departments corroborates this drastic decline in RSV cases (Figure 2A). In contrast, rhinovirus detections were well above average.

Panel A: 3-week moving average for bronchiolitis, a clinical proxy for RSV. Source: Public Health Rapid, Emergency, Disease and Syndromic Surveillance (PHREDSS) system, Centre for Epidemiology and Evidence, NSW Ministry of Health.

Panel B: Respiratory viruses tested were influenza, RSV and rhinoviruses. Lines show the 3-week moving average for each virus. Grey bars show the total tests performed per week (right y-axis). Source: New South Wales Health Pathology.

Panel C: Respiratory viruses tested were influenza, RSV and rhinoviruses. Grey bars show the total tests performed per week (right y-axis). Source: PathWest Laboratory Medicine, Perth.

The state’s capital, Perth, where most of the population resides, is ca 3,000 km from the NSW capital, Sydney, yet the patterns of virus percent-positive are very similar in the two cities.

To enable total case finding for COVID-19, the threshold for respiratory virus testing has declined, with the broadening of case definitions and indications for testing, irrespective of the presence of symptoms. These minimal criteria may explain the very high percentage of detections positive for rhinovirus infection in NSW and WA since around week 22 (Figure 2). It is possible that these infections were detected in people who may not have met clinical criteria for testing in prior years. Alternatively, absent competition from influenza and RSV [8], rhinoviruses may be taking advantage of this niche. The drop in rhinovirus percentage-positivity around weeks 15–21 coincided with school holidays, 4-week school closures in NSW and voluntary withdrawal of children from school by many parents, underscoring the role of children in rhinovirus circulation [9].

## Discussion

The very low numbers of influenza virus detections since April raises the possibility that some of the notified infections may be false positive. Among 10 influenza viruses (6.7% of all notified by diagnostic laboratory testing) received in August and September at the WHO Collaborating Centre for Reference and Research on Influenza in Melbourne, none could be confirmed as influenza by real-time RT-PCR or culture. Given that the surveillance data suggested a prevalence < 0.05% [10], a test with 95% sensitivity and 99.99% specificity would still only be expected to have a positive predictive value of 82.6%. This means that at least 15% of samples currently being diagnosed as influenza positive may be false positive.

Australia’s annual influenza vaccination campaign commenced in April 2020. Vaccination, funded under the National Immunisation Program, was provided free of charge to many high-risk individuals [11,12] and all Australians were strongly encouraged to become vaccinated to alleviate any unnecessary burden on healthcare services should SARS-CoV-2 and influenza viruses co-circulate. Vaccine supply was sufficient to cover 70% of the eligible population and meet heightened demand in 2020 [13]. ILI surveillance data suggested uptake increased from 27% in 2019 to 45% in 2020. Online ILI surveillance data available through Flutracking [14] suggested that up to 93% of healthcare workers were vaccinated this year, an increase from 88% in 2019. While it is possible that vaccination may have made a small contribution to reducing the spread of influenza, distinguishing the relative contribution of vaccination from that of physical distancing, movement and travel restrictions, hand hygiene and other measures is difficult.

We have previously shown that influenza epidemics in major Australian cities are highly synchronous, with frequent co-circulation of viruses from genetic subgroups in cities up to 3,000 km apart, suggesting significant mixing of the national population [15]. The closure of inter-jurisdictional borders appears to have effectively limited this mixing. In addition, genomic data suggest that influenza virus circulation in Australia usually involves multiple introductions from the global influenza virus population [15]. The drastic reduction in international travel and the mandatory 14-day quarantine in hotels on entry—which is sufficiently long and stringent for respiratory infections to resolve—as well as low circulation of influenza globally [16], has limited potential new virus introductions.

Like Australian jurisdictions, European countries frequently observe coinciding influenza seasons [17] that are dependent on introductions from other regions [18]. As at mid-November 2020, European sentinel surveillance indicates extremely low levels of influenza virus activity [19]. The limited number of influenza viruses currently circulating may have minimal opportunities to seed seasonal epidemics in Europe, as they are likely to be severely constrained by the recent re-implementation of restrictions on movement and mixing within the continent, as well as heavily reduced air travel to Europe.

## Conclusions

The COVID-19 pandemic and related mitigation strategies have exerted a strong impact on the circulation of influenza, RSV and other respiratory viruses. Globally, influenza viruses are in a severe bottleneck, complicating influenza vaccine decision-making for the upcoming seasons. Recent circulation of influenza A(H3N2) viruses in Cambodia and Bangladesh from July to September highlights the potential for the re-introduction of annual epidemics once global travel resumes. Recent increases in RSV activity in NSW and South Africa [20] suggest that RSV recirculation may precede that of influenza. It could be some time before global respiratory pathogen circulation returns to normal levels. In the meantime, the 2020 pandemic restrictions may substantially ameliorate the winter respiratory pathogen epidemics in 2021 and beyond. Compared with many European countries, the pandemic control response in Australia has comprised more stringent border control and stricter rules to limit social mixing. Nevertheless, interventions used to limit person-to-person transmission of SARS-CoV-2 are the same as those that would be recommended in an influenza pandemic and should therefore be expected to limit the spread of seasonal influenza as well. Low circulation of influenza virus is already apparent in Europe [19], but whether this continues remains to be seen.
