# Erratum for Euro Surveill. 2020;25(47)

**DOI:** 10.2807/1560-7917.ES.2021.26.21.210527e

**Published:** 2021-05-27

**Authors:** Eurosurveillance editorial team

**Affiliations:** 1 European Centre for Disease Prevention and Control (ECDC), Stockholm, Sweden

In the article ‘Where has all the influenza gone? The impact of COVID-19 on the circulation of influenza and other respiratory viruses, Australia, March to September 2020’ by Sullivan et al. published on 26 November 2020, the date period 2015–2019 was incorrectly written as 2015–2109 in the abstract. This mistake was corrected on 21 May 2021. We apologise for any inconvenience this typo may have caused.

